# In remembrance of David Yaffe

**DOI:** 10.1186/s13395-020-00246-1

**Published:** 2020-10-24

**Authors:** Margaret Buckingham, Eldad Tzahor

**Affiliations:** 1 CNRS UMR3738, Department of Developmental and Stem Cell Biology, Pasteur Institute, Paris, France; 2 Department of Molecular Cell Biology, Weizmann Institute of Science, Rehovot, Israel


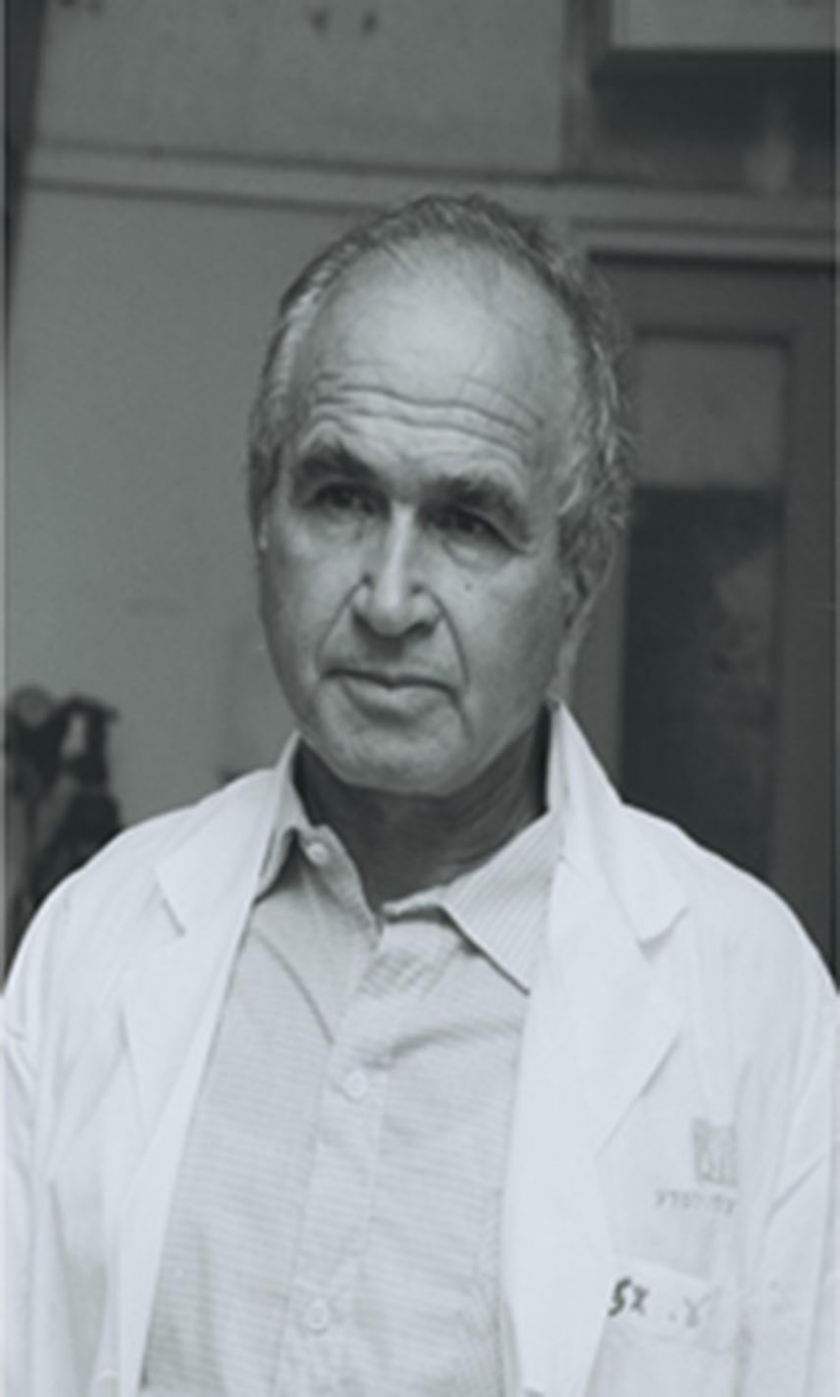


The muscle community is mourning the loss of David Yaffe, emeritus professor at the Weizmann Institute of Science in Israel, who died at the beginning of July. His pioneering work on muscle cell biology played a major role in founding the modern field of myogenesis.

David was born in 1929 in Tel Aviv. He was passionate about the nature of life and focussed his studies on biology and agriculture. However in 1948, he put aside his academic training to join the Palmach, the underground military organisation that fought for the establishment of an independent state of Israel. MB remembers how he regarded her with some suspicion as a representative of the enemy colonial power until he realised that her Scottish origins meant that her ancestors had also fought the English for their freedom! David was a firm believer in the principles of the founding fathers of Israel and lived all his life on a kibbutz, with his wife and family.

In 1952 he started his M.Sc. studies in Biology at the Hebrew University of Jerusalem and after graduating went on to do a Ph.D. with Michael Feldman in the Cell Biology department of the Weizmann Institute. In 1961, he began his career there as an independent researcher. Apart from a sabbatical year in Robert Schimke’s laboratory at Stanford, he spent his entire career in the Weizmann Institute - over six decades.

Already at the onset of his research career, David realised the need for a system that would permit the study of developmental processes outside the embryo. He recognised the potential of skeletal muscle where muscle precursor cells, myoblasts, are able to differentiate and fuse together to form muscle fibers. The morphological distinction between mononucleated myoblasts and multinucleated fibers makes it possible to physically separate them, with the possibility of culturing the myoblasts. This culture system was being developed with chick muscle; David’s major contribution was to develop it using mammalian cells, first with primary cultures obtained from newborn rat muscle where he showed that proliferating myoblasts would grow as a monolayer in culture and that, when grown in a medium that was less rich in growth factors, these cells would differentiate into muscle fibers, thus demonstrating their intrinsic capacity to maintain muscle identity.

He went on to develop muscle cell lines, first from rat, such as the L6 line (isolated in his lab during the Six-Day War in 1967) [1, 2] and then, 10 years later, from mouse muscle. Based on his experimental observations of muscle cell behaviour, he developed conceptually important views about cell differentiation and tissue identity. He communicated his muscle cell expertise and made his cell lines available to others. The C2 mouse muscle line [3] and its subclone C2C12, has provided, and continues to provide, a major tool for many researchers working on myogenesis.

The Yaffe lab went on to characterise the molecular changes that occurred at the time of differentiation, in terms of the synthesis of muscle contractile proteins and of their messenger RNAs. It is hard to imagine this now, but prior to the advent of DNA cloning, it was only possible to follow radioactive labelled RNA [4] and to identify the presence of specific messenger RNAs by in vitro translation followed by SDS gel analysis of muscle contractile proteins [5]. In the 1970s, MB, working in François Gros’ lab at the Pasteur Institute in Paris, was also carrying out this type of analysis on muscle cell cultures. She remembers well David’s help with setting up the culture system, and also his interest in comparing results. There was a tinge of competition between the labs, but for David the science always came first. Subsequently with cloned probes [6] it became possible to look precisely at messenger RNAs and the transcriptional changes that took place at the time of differentiation [7]. MB began to focus on myogenesis in vivo using the mouse embryo, while the Yaffe lab continued to make important contributions with the in vitro model of myoblast culture.

Further reinforcing their findings on transcriptional regulation, the Yaffe lab demonstrated that DNAase1 hypersensitivity of muscle genes correlates with their expression at the time of cell fusion [8]. They also characterised muscle contractile protein genes [9] and their chromosomal location [10] and went on to look at their regulation [11]. In addition to their work on the transcriptional control of muscle cell differentiation, the Yaffe lab also examined the cell biology of the system and provided early insights into the manipulation of fusion and differentiation, for example by altered Ca^2+^ levels [12].

In a later phase of his research, David with Uri Nudel became interested in the Dystrophin gene which, when mutated, leads to Duchenne Muscular Dystrophy. In addition to its expected expression in differentiated myogenic cell cultures, they also showed that the gene is expressed in the brain [13]. They went on to characterise the promoters involved [14]. They also identified other transcripts from the Dystrophin gene and notably the Dp17 transcript controlled by a non-muscle specific promoter [15], which they then characterised functionally [16].

After his “retirement” David continued to be scientifically active, participating in work on Dp17 and Utrophin and also pursuing an interest in the stem cell properties of muscle cells, as well as in their manipulation to promote tissue regeneration, as evidenced by a paper published in his nintieth year [17].

In addition to his own research contributions, David Yaffe had major impact in federating the myogenesis research community. He understood the importance of informal communication of experimental results and ideas between the initially small number of researchers in the world working in what was then an emerging field. To promote this, he organised the first workshop on the subject in 1975 at the kibbutz of Shoresh near Jerusalem. This was followed by a second meeting there in 1980, where the strong personalities of Howard Holtzer and Irvin Konigsberg, together with David the great men of muscle cell culture, led to clashes of opinion, which David managed to deflect from confrontation to constructive discussion. In the following years, David organised other myogenesis meetings, notably in Ein Gedi. They were memorable for the science and also for what David showed us of the history and natural beauty of Israel. This tradition of international workshops supported by EMBO, initiated by David, and subsequently organised in many European countries, continues today to the great benefit of a now much larger research community interested in muscle formation and regeneration. The last such meeting where David was present was at the Weizmann Institute in 2016. He was invited, as an honoured participant, to give an evening talk. He spoke of his beloved muscle cells but also read poems he had written, translated from Hebrew, with accompanying photographs of the local countryside and its birds and flowers.

David Yaffe was passionately interested in science, and loved to discuss scientific ideas. He was tenacious and sometimes irrascible, but always true to his ideal of the pursuit of knowledge. As an experimental scientist he was rigorous and cautious about drawing rapid conclusions. When he published results they were well verified and the interpretation reliable. He was kind and encouraging to young scientists and welcomed newcomers to the field. In addition to his scientific interests, David was very knowledgeable about the archaeology, history and natural history of Israel. He was also interested in art and would try to combine a visit to an exhibition with a scientific trip abroad. Prized catalogues would be taken back to be read before they were given to the kibbutz library. David lived most of his adult life in the Kibbutz Givat Brener, a collective social community near Rehovot, that was traditionally based on agriculture. He was passionate about certain plants and special fruit trees that he grew in his garden. In addition to his remarkable scientific legacy, he leaves behind his family, his wife Ruth, three children and nine grandchildren.

When ET informed the myogenic community of David’s death, many colleagues wrote tributes to his memory. Extracts from some of these are presented here. Stephen Tapscott (Fred Hutchinson Cancer Research Center, Seattle) emphasises David’s conceptually important contribution to understanding lineage commitment, with the muscle cell systems he developed. He cites key findings in the 1960s – “the specificity of myogenic fusion between muscle cells and not other cell types; the irreversibility or “stability” of muscle differentiation and its implications for cancer; the retention of myogenic differentiation potential during prolonged myoblast replication in culture”. In addition to these early scientific insights, the cell lines that David established provided a valuable tool for many investigators. Michael Rudnicki (Ottawa Hospital Research Institute) wrote “David was without question one of the fathers of the modern myogenesis field. He was a gifted experimentalist whose many contributions form the foundation of our area of study. Notable was his derivation of C2 cells which facilitated and accelerated the cell and molecular investigation of myogenesis and continued to do so to this day.” Eric Olson (University of Texas, Southwestern Medical Center, Dallas) also emphasises this contribution “Among his many achievements was the establishment of the C2 cell line which enabled the discovery and analysis of MyoD, MEF2 and other myogenic factors and elucidation of the mechanisms of muscle gene regulation.” Andrew Lassar (Harvard Medical School) underlines the importance of this cell line for his discovery of MyoD, “In my own case the phenotypic stability of C2C12 cells (derived C2 cells) provided a great reality check to ensure that the search for a muscle determinant (i.e. MyoD) in azacytidine–induced 10T1/2 myoblasts (which displayed great phenotypic variation) would have some physiological relevance.”

The importance for the field of the myogenesis meetings that David initiated was also highlighted, with memories of the first meetings in Israel. Frank Stockdale (Stanford University) wrote “Because of David, the early meetings at Shoresh and elsewhere established Israel as a central facilitator for transfer of information, collaboration, and the social-scientific structure so important in our field. David tirelessly fostered these and the conferences that subsequently took place around the world. We are indebted to David for this, because without his efforts all of us would have had a harder time developing our careers”. Helen Blau (Stanford University) recalled “David Yaffe gave me my first big opportunity to give a talk at an international muscle meeting he organised in Shoresh, Israel. There were four women speakers, which was revolutionary at that time. That meeting made a deep impression on me – and launched me in the field.” She also comments “.. during the meeting we hiked the Wadi Qelt in spring, amidst wild flowers in full bloom, a miracle of beauty..” . Steve Hauschka (University of Washington Medical School, Seattle) adds “My fondest Shoresh memory is David offering to lead me into a small nearby forest to see wild Cyclamen plants blooming. I don’t recall his exact words, but our walk became akin to a pilgrimage for David as the delicate flowers were in some way enshrined in memories of his youth and the 1948 conflict – perhaps the contrast of wartime horrors and the Cyclamen’s enduring peaceful beauty”.

Following on from Helen Blau’s comment, others also stressed David’s encouragement of young researchers. Simon Hughes (MRC/King’s College, London) wrote “David was always an enthusiast and always happy to chat with any young and unknown postdoc whenever he got the chance. To me, he helped define the warmth and collegial nature of the myogenesis field. Many young scientists need the kind of encouragement he gave.” Also David Sassoon (UCSF and VA Hospital, San Francisco; INSERM PARCC, Paris) wrote “He was able to critique work without being condescending and at meetings would spend much time with students at their posters and with young colleagues just starting out in their professional lives.” Mary Baylies (Sloan Kettering Institute, New York) adds to this “I was scheduled to give a talk (my first!) at a Myogenesis meeting. After the talk David sought me out. He let me know that he was sceptical at the beginning of my talk but at the end he was convinced – both of the use of Drosophila as a model to dissect myogenesis and my ability to give a talk! … His wonderful presence and ability to reach out to junior colleagues in the field created a great atmosphere in our community.”

A final comment cited here came from Barbara Wold (California Institute of Technology, Pasadena) on David’s personality and capacity to attract newcomers to the field. “My first thought is of David as a wonderful, stubborn, positive force for the entire field. He leaves a lasting legacy that flows from his specific science contributions and also from his force of personality. His curiosity and interest never flagged through the decades. He was intellectually and personally gracious, and especially encouraging of scientists newly interested in myogenesis - whatever their age or career stage. A scientific argument with David was the fun kind of argument from which I usually learned something - win, lose, or draw. He was welcoming of people like me and my students who arrived as newcomers from other fields and scientific traditions. We were not treated as strangers - but rather as new recruits into the myogenesis brigade. In this and many other ways, David helped to build a vibrant and always evolving community.”

Other tributes and reminiscences about David Yaffe, mostly from previous collaborators who had worked in his lab, have been assembled by Zippora Yablonka-Reuveni (University of Washington School of Medicine, Seattle) and can be found at:

Yablonka-Reuveni, Z., Stockdale, F., Nudel, U., Israeli, D., Blau, H. M., Shainberg, A., Neuman, S., Kessler-Icekson, G., Meghid Krull, E., Paterson, B., Saxel Fuchs, O., Greenberg, D., Sarig, R., Halevy, O., Ozawa, E., & Katcoff, D. J. (2020). Farewell to Professor David Yaffe – A Pillar of the Myogenesis Field. European Journal of Translational Myology. 10.4081/ejtm.0.9306
